# Far infrared radiation promotes rabbit renal proximal tubule cell proliferation and functional characteristics, and protects against cisplatin-induced nephrotoxicity

**DOI:** 10.1371/journal.pone.0180872

**Published:** 2017-07-17

**Authors:** I-Ni Chiang, Yeong-Shiau Pu, Chao-Yuan Huang, Tai-Horng Young

**Affiliations:** 1 Institute of Biomedical Engineering, College of Medicine and College of Engineering, National Taiwan University, Taipei, Taiwan; 2 Department of Urology, National Taiwan University Hospital, College of Medicine, National Taiwan University, Taipei, Taiwan; 3 Department of Urology, National Taiwan University Hospital, Hsin Chu Branch, Hsin Chu City, Taiwan; University of PECS Medical School, HUNGARY

## Abstract

Far infrared radiation, a subdivision of the electromagnetic spectrum, is beneficial for long-term tissue healing, anti-inflammatory effects, growth promotion, sleep modulation, acceleration of microcirculation, and pain relief. We investigated if far infrared radiation is beneficial for renal proximal tubule cell cultivation and renal tissue engineering. We observed the effects of far infrared radiation on renal proximal tubules cells, including its effects on cell proliferation, gene and protein expression, and viability. We also examined the protective effects of far infrared radiation against cisplatin, a nephrotoxic agent, using the human proximal tubule cell line HK-2. We found that daily exposure to far infrared radiation for 30 min significantly increased rabbit renal proximal tubule cell proliferation in vitro, as assessed by MTT assay. Far infrared radiation was not only beneficial to renal proximal tubule cell proliferation, it also increased the expression of ATPase Na+/K+ subunit alpha 1 and glucose transporter 1, as determined by western blotting. Using quantitative polymerase chain reaction, we found that far infrared radiation enhanced *CDK5R1*, *GNAS*, *NPPB*, and *TEK* expression. In the proximal tubule cell line HK-2, far infrared radiation protected against cisplatin-mediated nephrotoxicity by reducing apoptosis. Renal proximal tubule cell cultivation with far infrared radiation exposure resulted in better cell proliferation, significantly higher ATPase Na+/K+ subunit alpha 1 and glucose transporter 1 expression, and significantly enhanced expression of *CDK5R1*, *GNAS*, *NPPB*, and *TEK*. These results suggest that far infrared radiation improves cell proliferation and differentiation. In HK-2 cells, far infrared radiation mediated protective effects against cisplatin-induced nephrotoxicity by reducing apoptosis, as indicated by flow cytometry and caspase-3 assay.

## Introduction

Infrared radiation is optical radiation from wavelength 780 nm to 1 mm, and the infrared region in the wavelength range of 3 μm to 1 mm is further defined as far infrared radiation (FIR) [1]. Recently, FIR, part of the electromagnetic spectrum, has been proven to be beneficial for long-term tissue healing, protection against inflammation, promotion of growth, modulation of sleep, acceleration of microcirculation, and pain relief [2]. The apoptosis and cell death of dehydration-stressed cultured keratinocytes was attenuated by FIR [3]. The biological influences of FIR on cells in vitro include effects on nitric oxide, calmodulin induction, intracellular heat shock protein, and intracellular nitric oxide contents in melanoma cells, skeletal muscle cells, and human breast epithelial cells [4]. Wounds treated with FIR healed more quickly, with better collagen regeneration and more fibroblast proliferation, than [5]. The tensile strength of the unwounded skin of rats exposed to FIR was significantly higher than the tensile strength of the unwounded skin of rats without FIR exposure over 2 wk [2]. In this study, we investigated if FIR could be beneficial in renal proximal tubule cell (RPTC) cultivation and renal tissue engineering.

RPTCs are the most important and fragile components of the renal cortex; they are responsible for 60% of kidney reabsorption of glomerular filtrations, including water, electrolytes (sodium, potassium, calcium, phosphate, and bicarbonate), glucose, and amino acids [6]. RPTC cultivation is an emerging field in tissue engineering, with possible future clinical applications for nephrotoxicity assays, regenerative medicine, and the construction of a bioartificial kidney [7]. Several studies have focused on optimizing RPTC cultivation via adjustments to the growth medium, serum supplements, digestion methods, extracellular matrix components, and biomaterials used for cultivation [8–11]. All living organisms experience the beneficial and side effects, from the level of transcription to the whole organism, of natural electromagnetic radiation [12]. As there have not been any studies on photomodulation and RPTC cultivation, we examined the effects of FIR on RPTCs, including effects on cell proliferation, functional characteristics, protein expression, and subcellular findings. We further investigated the protective effects of FIR against the nephrotoxic agent cisplatin on the human proximal tubule cell line HK-2.

## Materials and methods

### Renal tissue retrieval

This animal study was performed with an animal use protocol approved by the Review Committee of Medical College, National Taiwan University. Adult, male, New Zealand white rabbits (body weight from 3 to 5 kg) were used in this study. Rabbits were anesthetized with an intramuscular injection of Rompun™ (xylazine, 0.1 ml, 25 mg/ml, ASC-King) and Zoletil® (1 ml, 50 mg/ml, ASC-King). If anesthesia was not satisfactorily achieved, a second shot of 0.1 ml and third shot of Rompun™ would be given. The rabbit was placed in the right flank position and a left subcostal oblique incision was made. The wound was deepened into the retroperitoneum until the left kidney was identified. The renal pedicle was restricted with 3–0 silk suture, then the left kidney was resected and put into Hank’s Balanced Salt Solution (HBSS). The wound was closed in layers with 3–0 nylon suture. Rabbits were sent to the National Taiwan University Laboratory Animal Center for recovery.

### Cell culture: Primary RPTCs and the HK-2 cell line

The renal tissue harvested from adult, male, New Zealand white rabbits was chopped, placed into HBSS containing 30 mg collagenase, and incubated at 37°C for 5 min. The fragments were filtered through a 125-m sieve, washed 3 times with fresh HBSS, centrifuged at 500 rpm for 5 min, and resuspended in Dulbecco’s Modified Eagle Medium: Nutrient Mixture F-12 (DMEM/F12, Gibco) with 3% bovine serum albumin (Sigma-Aldrich) at 4°C for 15 min. RPTCs were further purified by centrifugation on 45% Percoll® (Pharmacia Biotech) at 14,500 rpm for 30 min at 4°C, washed 3 times with HBSS, and resuspended in culture medium that consisted of equal parts DMEM/F12 (Gibco) and Keratinocyte Serum-Free Medium (Gibco), supplemented with 50 g/ml bovine pituitary extract (Gibco); 10 ng/ml epidermal growth factor (Gibco); 5 g/ml insulin, 5 g/ml transferrin, 5 ng/ml selenium (I-T-S, Gibco); 10^−3^ M hydrocortisone; 0.5% dimethyl sulfoxide (Sigma-Aldrich); and 1% antibiotics (Gibco). Type I collagen (BD Biosciences)-coated culture plates were used for cell cultivation. RPTCs were maintained at 37°C in a humidified atmosphere of 5% CO_2_ in 95% air, and the culture medium was changed every 2 d.

The HK-2 cell line was purchased directly from Bioresource Collection and Research Center (BCRC, Hsinchu, Taiwan) and grown in Defined Keratinocyte Serum-Free Medium (Gibco) containing 40 μg/ml bovine pituitary extract and 1% antibiotics (Gibco) at 37°C in a humidified 5% CO_2_ incubator. The medium was changed every 2 to 3 d.

### FIR exposure

A ceramic FIR generator, the WS TY301 FIR emitter (WS Far Infrared Medical Technology), was used to provide FIR exposure. The FIR emitter generates electromagnetic waves with wavelengths in the range of 3 to 25 μm. RPTCs were cultured in 24-well culture plates and divided into the FIR group (with FIR exposure) and the control group (without FIR exposure), each in quadruplicate, for further comparison. The FIR group was placed on the axis of the emitter at a distance of 30 cm for 30 min of daily FIR exposure, according to a published protocol [3, 4].

### Cell proliferation assessment

The proliferation of RPTCs with and without FIR exposure was assessed, in 4 independent experiments, by evaluating their mitochondrial activity with a 3-(4,5-dimethylthiazol-2-yl)-2,5-diphenyltetrazolium bromide (MTT) assay (Sigma-Aldrich) [5]. Day 0 was defined as the date of cell seeding. MTT was prepared as a 5 mg/ml stock solution in phosphate-buffered saline (PBS), sterilized by Millipore filtration, and protected from light. MTT solution (50 μl) was added to each well without the removal of the culture medium. After 3 h of incubation at 37°C, 200 μl of dimethyl sulfoxide was added to dissolve the formazan crystals. The solution was then agitated for approximately 15 min on a shaker. The optical density of the formazan solution, for quadruplicate samples, was read at 570 nm on an ELISA plate reader (ELx800, BIO-TEK).

### Immunofluorescence microscopy

The primary antibodies used in this study included rabbit polyclonal antibodies against ATPase Na+/K+ subunit alpha 1 (Abcam) and glucose transporter 1 (GLUT1). For immunostaining, cells were washed 3 times with PBS, fixed with 4% paraformaldehyde for 15 min at room temperature, then permeabilized with 0.1% Triton™ X-100. After they were blocked with 3% bovine serum albumin in 0.05% Tween® 20 in PBS, the cells were stained with the indicated primary antibody at 4°C overnight. The cells were then washed and incubated with the appropriate Alexa Fluor® 488-conjugated secondary antibodies, and counterstained with 4',6-diamidino-2-phenylindole. Finally, fluorescence images were acquired using a ZEISS Axio-200 inverted microscope and analyzed by MetaMorph® software (Molecular Devices).

### Western blot analysis

Cell lysates were incubated in RIPA lysis buffer containing cOmplete™ Protease Inhibitor Cocktail (Roche Diagnostics) for 1 h at 4°C. Lysates were clarified by centrifugation at 10,000 rpm for 10 min at 4°C and the resulting supernatants were saved for protein concentration and western blot analysis. Protein concentrations were measured using a commercial protein assay reagent (Bio-Rad). An equal volume of each sample was loaded into a 12% gel for sodium dodecyl sulfate polyacrylamide gel electrophoresis and transferred onto polyvinylidene fluoride membranes. The membranes were blocked with CISblock buffer (CIS-Biotechnology) for 1 min. Antibodies against ATPase Na+/K+ subunit alpha 1 and GLUT1 were used as primary antibodies. Horseradish peroxidase-conjugated secondary antibodies were purchased from Millipore. Antigen–antibody complexes were visualized by enhanced chemiluminescence (Millipore). The results were analyzed from quadruplicate samples.

### Quantitative real-time polymerase chain reaction (qPCR)

Briefly, total RNA was extracted from RPTCs using TRIzol® reagent (Invitrogen Life Technologies), according to the manufacturer's instructions. Next, 5 μg of total RNA was used to prepare cDNA. A reverse transcriptase kit (Superscript® III, Invitrogen Life Technologies) was used for cDNA synthesis, at 55°C for 50 min, followed by 85°C for 5 s, on an ABI GeneAmp® PCR System 9700 (Applied Biosystems). The transcripts were quantified using the ABI 9700 and SYBR® Premix reagent (Thermo Fisher Scientific), according to the manufacturer's instructions. Reactions began with a 10-s hot-start activation of the Taq polymerase at 95°C, followed by 45 cycles of amplification in 3 steps (denaturation at 95°C for 5 s, followed by 30 s annealing at 60°C, and 30 s extension at 72°C). The primers used in each reaction are shown in Table 1. Data are expressed as the mean ± standard deviation of 4 independent experiments.

**Table 1:** Primers used for specific PTC genes expression.

Target genes	Description	Primer sequences
*ATP5B*	ATP synthase H+ transporting mitochondrial F1 complex beta polypeptide	F: 5’-ATGGATGGCACAGAGGGCTT-3’ R: 5’-TTTGATGGGACCTCGCTCGT-3’
*CDK5R1*	cyclin-dependent kinase 5 regulatory subunit 1	F: 5’-AAGAACCTGAAGCGGCACT-3’ R- 5’-GTGCGTGATGTTGTTCTGGT-3’
*GNAS*	GNAS complex locus	F: 5’-AGGACAACCAGACCAACCGT-3’ R: 5’-TTCTCAGCCAGCAGGTCTTG-3’
*NPPB*	natriuretic peptide B	F: 5’-TCTCCTGCTCCTCCTCTTCTT-3’ R: 5’-TGGGAGACCTTGTTCCGGAG-3’
*TEK*	TEK tyrosine kinase endothelial	F: 5’-GGCTCCTTTATCCATTCGGTG-3’ R: 5’-GCCGAAGTGAAGAGGTTTCCT-3’
*GAPDH*	Glyceraldehyde 3-phosphate dehydrogenase	F: 5’- TCACCATCTTCCAGGAGCG -3’ R: 5’- GAGATGATGACCCTTTTGGC -3’

### Assessment of protection against cisplatin-induced nephrotoxicity

HK-2 cells (10^5^) were seeded in a 24-well plate (Day 0). The HK-2 cells were grouped for cultivation with and without FIR, defined as the FIR and control groups, respectively, on Day 1. The FIR group received FIR exposure for 1 h. Then, cisplatin (50 μM) was added to the control and FIR cultures, and the cells were cultivated with cisplatin for 3 h. The cisplatin medium was then replaced with cisplatin-free medium. We performed an MTT assay and FITC Annexin V apoptosis staining on Day 2. The MTT assay was performed as described under Cell proliferation assessment. For the detection of apoptosis, the cells in each well were retrieved by digestion with 1 ml trypsin for 5 min at 37°C, followed by the addition of 4 ml medium, and centrifugation at 1,000 rpm for 5 min. The cell pellet was resuspended in 1 ml PBS. A 100-μl aliquot of cell suspension was placed in a 5-ml test tube. We sequentially added 5 μl of FITC Annexin V and 10 μl propidium iodide solution into the test tube. The cells were vortexed and incubated for 15 min at room temperature. Then, 400 μl of Annexin V Binding Buffer was added into each tube and quadruplicate samples were analyzed by flow cytometry.

### Caspase-3 assay

HK-2 cells were suspended in 50 μl of chilled Cell Lysis Buffer, incubated on ice for 10 min, and centrifuged for 1 min in a microcentrifuge (10,000 x *g*). Then, the supernatant was transferred to a fresh tube and stored at −80°C for future use. We diluted 50 μg protein in 50 μl Cell Lysis Buffer for each assay. We added 50 μl of 2× Reaction Buffer (containing 10 mM dithiothreitol) to each sample. We added 5 μl of 4 mM DEVD-pNA substrate (200 μM final concentration) and incubated the samples at 37°C for 1 h. Quadruplicate samples were read at 400 nm in a microtiter plate reader.

## Results

### Cell proliferation

**Fig 1:**
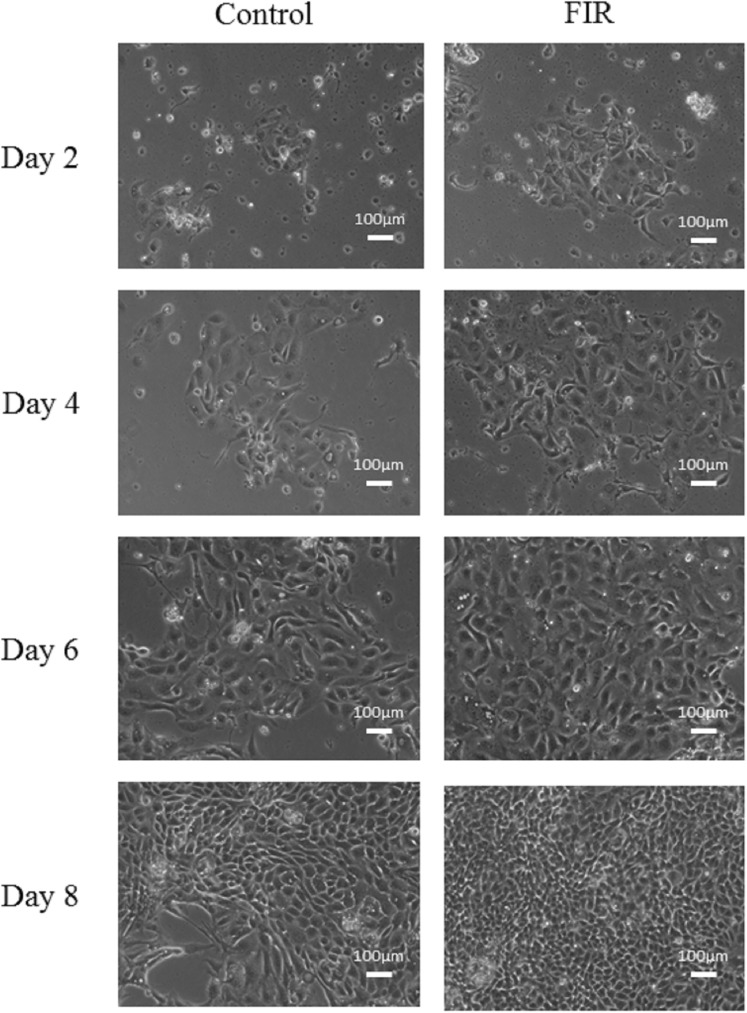
The morphology of RPTCs cultivated with or without FIR exposure. The FIR group comprises RPTCs subjected to daily, 30-min FIR exposure during cultivation. The control group comprises RPTCs cultivated without FIR exposure. Cell proliferation appeared similar in the control and FIR groups on Days 2 and 4, whereas it appeared higher in the FIR group from Day 6.

**Fig 2:**
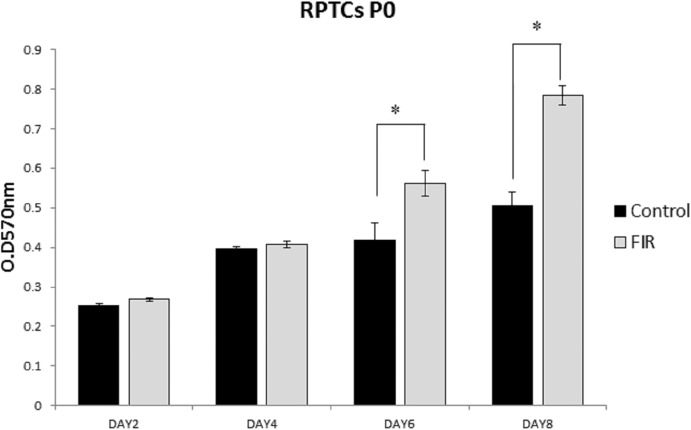
Exposure to FIR increases renal cell proliferation. Mitochondrial activity, assessed by MTT assay, was significantly higher in the FIR group than in the control group from Day 6 (p<0.05).

After enzymatic digestion, RPTCs were seeded on collagen-coated culture plates and cultivated with or without FIR. The cell morphology of the RPTCs in the control and FIR groups is presented in Fig 1. Qualitatively, cell proliferation appeared similar in both groups on Days 2 and 4, but it was higher in the FIR group from Day 6. The MTT assay revealed that mitochondrial activity, which indicates cell proliferation, was significantly higher in the FIR group than in the control group, starting on Day 6 (p<0.05, Fig 2).

### Immunofluorescence microscopy and western blot analysis

**Fig 3:**
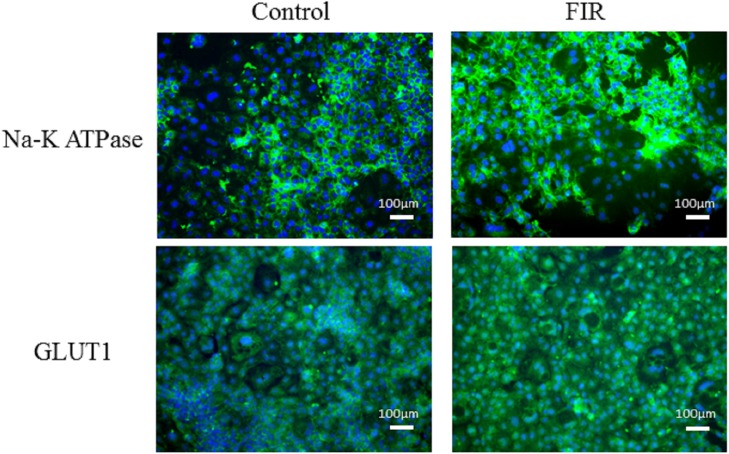
RPTCs exposed to FIR have more intense immunofluorescence staining for ATPase Na+/K+ subunit alpha 1 and GLUT1. RPTCs from the control and FIR groups were stained with antibodies against ATPase Na+/K+ subunit alpha 1 and GLUT1. The FIR group had qualitatively more intense staining for ATPase Na+/K+ subunit alpha 1 and GLUT1.

**Fig 4:**
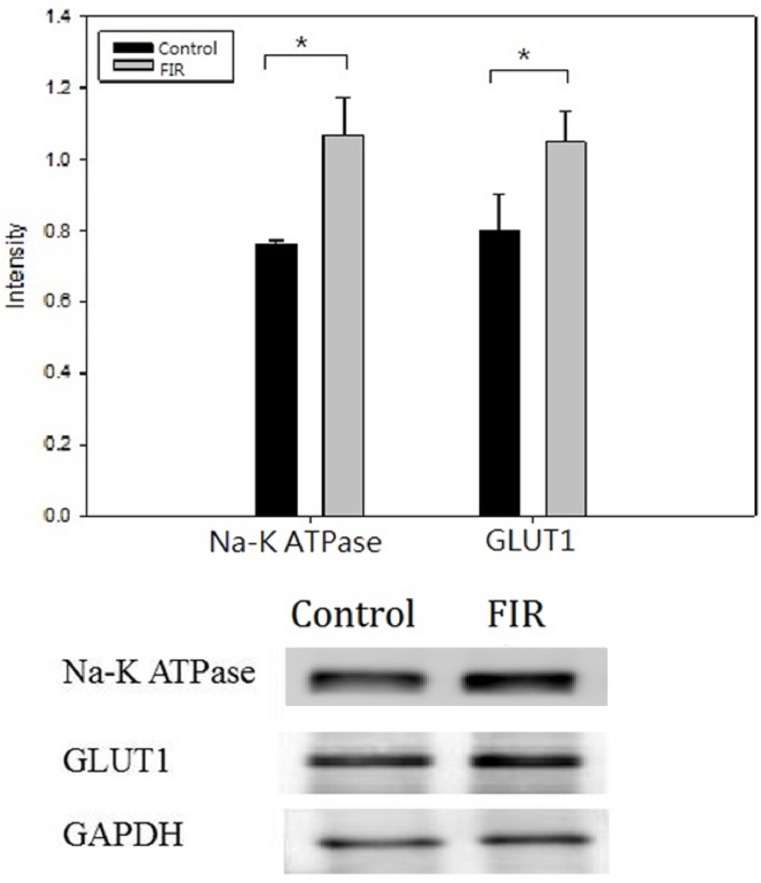
FIR exposure increases ATPase Na+/K+ subunit alpha 1 and GLUT1 expression. The FIR group had significantly higher Na^+^/K^+^ and GLUT1 expression, as assessed by western blotting, than the control group (p<0.05).

We stained the RPTCs from the control and FIR groups with antibodies against ATPase Na+/K+ subunit alpha 1 and GLUT1. Cells in both the control and FIR groups displayed the typical characteristics of RPTCs, with high ATPase Na+/K+ subunit alpha 1 and GLUT1 expression (Fig 3). Qualitatively, ATPase Na+/K+ subunit alpha 1 and GLUT1 staining were more intense in the FIR group. Western blot analysis with quantification confirmed that the FIR group expressed significantly higher levels of ATPase Na+/K+ subunit alpha 1 and GLUT1 than the control group (p<0.05, Fig 4).

### qPCR

**Fig 5:**
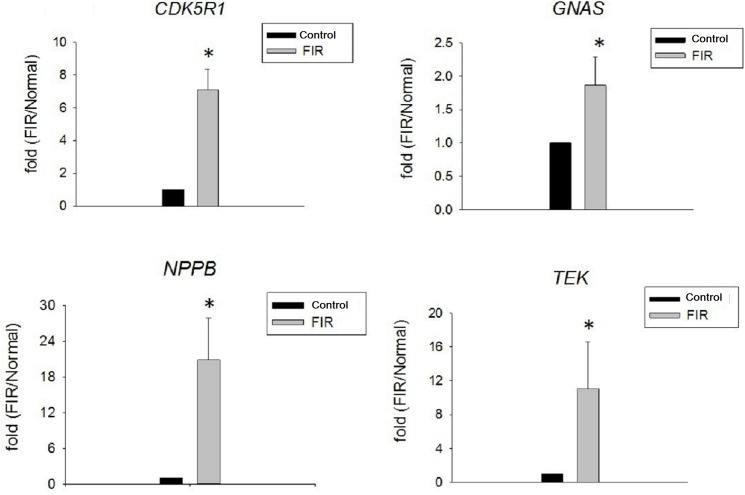
FIR exposure enhances the expression of *CDK5R1*, *GNAS*, *NPPB*, and *TEK* in RPTCs. We compared the expression of the indicated genes in the FIR group and the control group by qPCR (p<0.05).

Several genes have been shown to correlate with FIR exposure and cell proliferation by microarray (Accession number: GSE98096). Thus, we chose 4 target genes, *CDK5R1*, *GNAS*, *NPPB*, and *TEK*, for analysis by qPCR. FIR exposure significantly enhanced the expression of the target genes *CDK5R1*, *GNAS*, *NPPB*, and *TEK* (p<0.01, Fig 5).

### Protection against cisplatin-induced nephrotoxicity

**Fig 6:**
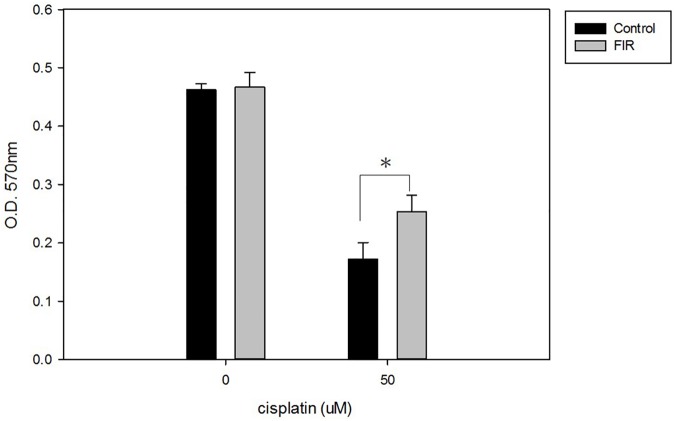
Exposure to FIR protects HK-2 cells from cisplatin-induced nephrotoxicity. On Day 2 of cisplatin treatment, HK-2 cells that had been exposed to FIR underwent a significantly higher degree of cell proliferation than the cells in the control group (p<0.05). Cell proliferation was assessed by MTT assay.

**Fig 7:**
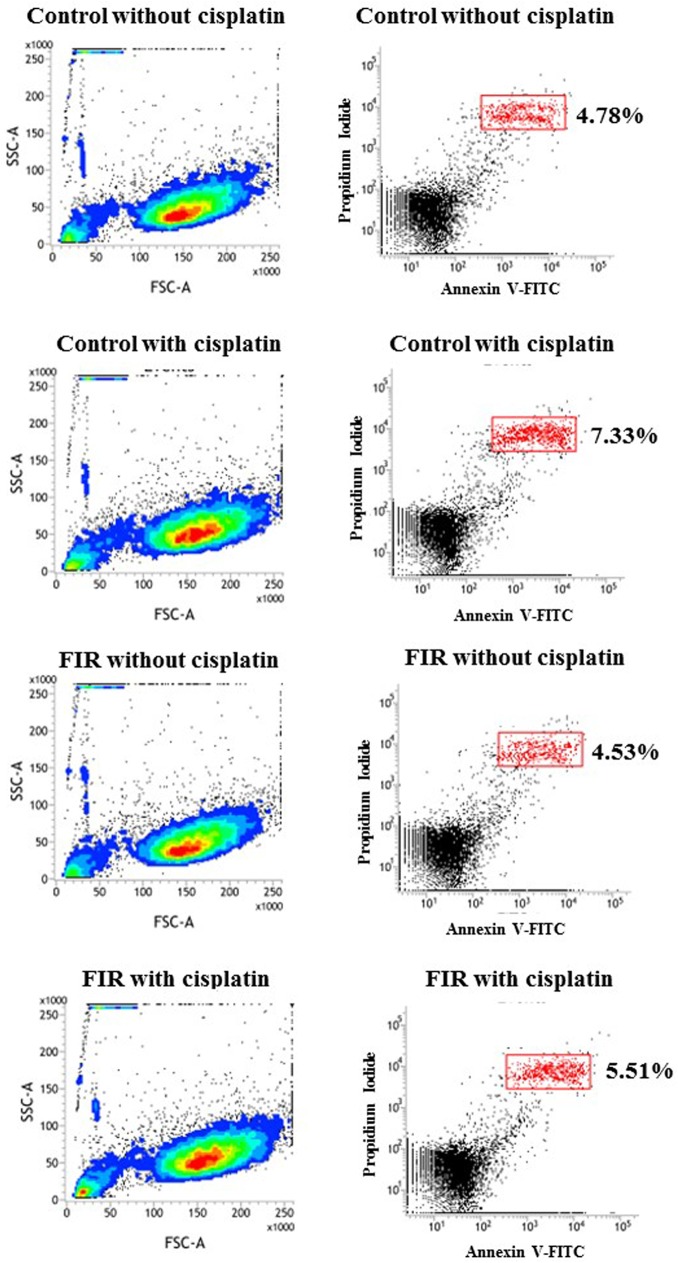
Cisplatin-induced apoptosis is blocked by FIR exposure in HK-2 cells. Cisplatin increased the rate of apoptosis in HK-2 cells. However, FIR exposure reduced the proportion of apoptotic HK-2 cells after cisplatin treatment in comparison with the control group.

**Fig 8:**
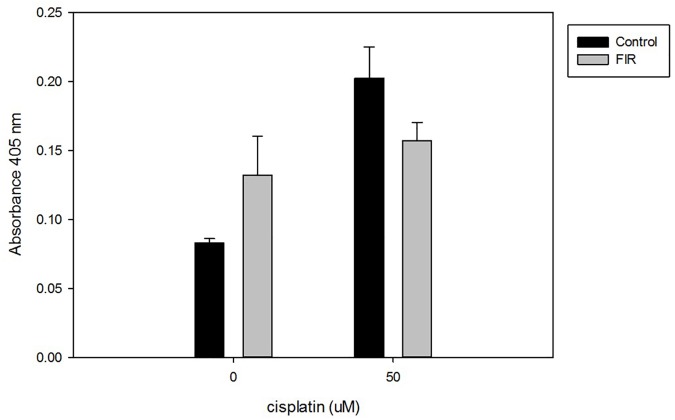
FIR exposure reduces caspase-3 activation. Using a caspase-3 activation assay, we found that caspase activity was higher in the control group than in the FIR group after cisplatin treatment.

The addition of cisplatin for 3 h significantly reduced cell viability in both the control and FIR groups. However, interestingly, cell viability remained significantly higher in the FIR group than in the control group, despite the nephrotoxicity caused by cisplatin (p<0.01, Fig 6). We observed higher levels of apoptosis upon cisplatin addition. However, HK-2 cells with FIR exposure had lower apoptosis levels than HK-2 cells without FIR (Fig 7). We next assessed caspase activity, which indicates apoptosis activation. Using a caspase-3 assay, we found that caspase activity was higher in the control group than in the FIR group after cisplatin addition (Fig 8).

## Discussion

FIR, using electromagnetic waves with a wavelength of 3 μm to 1 mm, has been reported to promote cell proliferation and increase tissue regeneration, as well as decrease wound healing time [13]. Our study showed that daily exposure to FIR for 30 min significantly increased RPTC viability in vitro, as assessed by MTT assay. FIR was not only beneficial for RPTC viability. RPTCs exposed to FIR expressed higher levels of ATPase Na+/K+ subunit alpha 1 and GLUT1. This statistically significant finding was documented by western blot analysis. We also found, by qPCR, that FIR significantly enhanced *CDK5R1*, *GNAS*, *NPPB*, and *TEK* expression. In HK-2 cells, FIR mediated protective effects against cisplatin-induced nephrotoxicity by reducing apoptosis.

In previous in vivo studies, FIR enhanced the wound healing rate by increasing the number of fibroblasts, enhancing collagen aggregation, and promoting TGF-β1 secretion [14]. FIR increased skin microcirculation via the L-arginine/NP pathway, exerting an NO-related biological effect that could be suppressed by N-nitro-L-arginine methyl ester (an endothelial nitric oxide synthase inhibitor); the effect was not influenced by atropine, propranolol, and phentolamine [15]. The previous literature mainly comprises in vitro studies that focused on keratinocytes and fibroblasts [3, 5]. This is the first study to assess the effects of FIR on RPTCs, which could contribute to further experiments on kidney tissue engineering and nephrotoxicity. In addition, we found that FIR is beneficial to RPTC viability. RPTCs subjected to daily FIR exposure for 15 min had significantly higher cell viability in the MTT assay than RPTCs without FIR.

Electromagnetic radiation influences all living organisms, by impacting water, DNA, proteins, cell fluids, cell membranes, subcellular organelles, and the whole organism. At the cell level, FIR can alter cell membrane potential and mitochondrial metabolism [12]. Using a preliminary microarray analysis, we selected several genes that correlated with FIR exposure for qPCR analysis; we found that FIR exposure significantly increased the expression of *CDK5R1*, *GNAS*, *NPPB*, and *TEK*. Cyclin-dependent kinase 5 (CDK5), a serine/threonine kinase identified 20 years ago, is associated with endothelial cell migration through the small GTPase Rac1 and nuclear factor-kappa B (NF-κB) [16]. Ma et al. found that activation of G-protein-coupled receptor 40 decreased cisplatin-induced RPTC apoptosis through inhibition of the generation of reactive oxygen species, the activation of the Src/EGFR/ERK signaling pathway, and NF-κB [17]. *GNAS* is a complex gene with multiple gene products, which show parent-of-origin-specific expression, including neurotransmitters, autocrine/paracrine factors, hormones, tyrosine kinases, and calcium channels [18]. Pseudohypoparathyroidism is one of the disorders associated with *GNAS* locus deficit, and the *GNAS* locus is associated with RPTC responses to parathyroid hormone and electrolyte balance [19–21]. Natriuretic peptides are naturally derived, cyclic peptide hormones that influence cardiovascular, endocrine, and renal homeostasis, and NPPB is essential for sodium excretion, water balance, and blood pressure regulation [22]. TEK is mainly expressed on endothelial cells [23]. FIR appears to enhance RPTC viability and increase the expression of *CDK5R1*, *GNAS*, *NPPB*, and *TEK*.

Yung et al. have reported protective effects of FIR on stressed keratinocytes that had been exposed to sorbitol; FIR pretreatment reduced dehydration-induced keratinocyte apoptosis and cell death [3]. We examined the protective effects of FIR against cisplatin-mediated nephrotoxicity on the human proximal tubule cell line HK-2. Before cisplatin addition, the FIR group were exposed to FIR for 1 h, whereas the control group was not exposed to FIR. After 3-h cisplatin treatment, we compared cell viability in the control and FIR groups. The FIR group had significantly higher cell viability than the control group.

Cisplatin is a well-known anticancer drug that induces a wide range of toxicities in a dose-dependent manner [24]. Cisplatin causes toxicity to auditory, renal, and neuronal cells by inducing the expression of active caspase-3, the nitration of proteins, and cell apoptosis 24 h post-treatment [25]. In our study, we also observed cisplatin-induced nephrotoxicity, as indicated by decreasing cell viability and increasing cell apoptosis in HK-2 cells. Since cisplatin is so widely applied for cancer treatment, many efforts have been made to reduce its toxic effects. Most studies have focused on medications to counteract cisplatin-mediated nephrotoxicity. In a rat model, 5-aminolevulinic acid dissolved in drinking water ameliorated cisplatin-induced morphological renal damage and reduced tubular apoptosis, as evaluated by terminal deoxynucleotidyl transferase dUTP nick end labeling and cleaved caspase-3 [26]. In another rat model, G-protein-coupled receptor 40, which plays an important role in fatty acid regulation, reduced cisplatin-induced kidney injury through the inhibition of reactive oxygen species generation, activation of the Src/EGFR/ERK pathway, and the nuclear activation of NF-κB [17]. Ours is the first study to investigate the protective effects of FIR against cisplatin-mediated nephrotoxicity to RPTCs through photomodulation. First, we observed that FIR increased RPTC viability during primary cultivation and enhanced ATPase Na+/K+ subunit alpha 1 and GLUT-1 expression. Our study also suggests that FIR not only enhances cell viability and the functional characteristics of RPTCs, but that it also protects against cisplatin-mediated nephrotoxicity by reducing apoptosis. FIR is a potential photomodulation therapy to facilitate RPTC cultivation that could possibly be applied in further studies on protection from nephrotoxicity and other cisplatin-mediated cell damage.

## Conclusions

RPTC cultivation with FIR exposure resulted in better cell proliferation, significantly higher ATPase Na+/K+ subunit alpha 1 and GLUT1 expression by immunofluorescence and western blot analysis, and significantly enhanced expression of *CDK5R1*, *GNAS*, *NPPB*, and *TEK* by qPCR. These results suggest that FIR exposure improves renal cell proliferation and differentiation. FIR also protected HK-2 cells against cisplatin nephrotoxicity by reducing apoptosis, as indicated by flow cytometry and caspase-3 activation.

## Supporting information

S1 File This is the raw data of Fig 2 in manuscript. Exposure to FIR increases renal cell proliferation.
(PDF)

S2 File This is the raw data of Fig 4 in manuscript. FIR exposure increases ATPase Na+/K+ subunit alpha 1 and GLUT1 expression.
(PDF)

S3 File This is the raw data of Fig 5 in manuscript. FIR exposure enhances the expression of CDK5R1, GNAS, NPPB, and TEK in RPTCs.
(PDF)

S4 File This is the raw data of Fig 6 in manuscript. Exposure to FIR protects HK-2 cells from cisplatin-induced nephrotoxicity.
(PDF)

S5 File This is the raw data of Fig 7 in manuscript. Cisplatin-induced apoptosis is blocked by FIR exposure in HK-2 cells.
(PDF)

S6 File This is the raw data of Fig 8 in manuscript. FIR exposure reduces caspase-3 activation.
(PDF)

## References

[1] International Commission on Non-Ionizing Radiation P. ICNIRP statement on far infrared radiation exposure. Health Phys. 2006;91: 630–45. doi: 10.1097/01.HP.0000240533.50224.65 17099407 10.1097/01.HP.0000240533.50224.65

[2] Yang CS, Yeh CH, Tung CL, Chen MY, Jiang CH, Yeh ML. Impact of far-infrared ray exposure on the mechanical properties of unwounded skin of rats. Exp Biol Med. 2010;235: 952–956. doi: 10.1258/ebm.2010.009372 20660095 10.1258/ebm.2010.009372

[3] Chen YC, Lai LC, Tu YP, Wu SD, Chen CF, Li B. Far infrared ray irradiation attenuates apoptosis and cell death of cultured keratinocytes stressed by dehydration. J Photochem Photobiol B. 2012;106: 61–68. doi: 10.1016/j.jphotobiol.2011.10.006 22062776 10.1016/j.jphotobiol.2011.10.006

[4] Leung TK. In Vitro and In Vivo Studies of the Biological Effects of Bioceramic (a Material of Emitting High Performance Far-Infrared Ray) Irradiation. Chin J Physiol. 2015;58: 147–155. doi: 10.4077/CJP.2015.BAD294 26014120 10.4077/CJP.2015.BAD294

[5] Toyokawa H, Matsui Y, Uhara J, Tsuchiya H, Teshima S, Nakanishi H, et al Promotive effects of far-infrared ray on full-thickness skin wound healing in rats. Exp Biol Med. 2003;228: 724–729. 10.1177/153537020322800612 12773705

[6] Shoskes D, McMahon AW. Renal Physiology and Pathophysiology. In: Campbell-Walsh Urology [Internet] Philadelphia: Elsevier Saunders 2012 pp. 10.

[7] Astashkina AI, Mann BK, Prestwich GD, Grainger DW. A 3-D organoid kidney culture model engineered for high-throughput nephrotoxicity assays. Biomaterials. 2012;33: 4700–4711. doi: 10.1016/j.biomaterials.2012.02.063 22444643 10.1016/j.biomaterials.2012.02.063

[8] Vinay P, Gougoux A, Lemieux G. Isolation of a pure suspension of rat proximal tubules. Am J Physiol. 1981;241: F403–411. 6119031 10.1152/ajprenal.1981.241.4.F403

[9] Vesey DA, Qi W, Chen X, Pollock CA, Johnson DW. Isolation and primary culture of human proximal tubule cells. Methods Mol Biol. 2009;466: 19–24. doi: 10.1007/978-1-59745-352-3_2 19148604 10.1007/978-1-59745-352-3_2

[10] Terryn S, Jouret F, Vandenabeele F, Smolders I, Moreels M, Devuyst O, et al A primary culture of mouse proximal tubular cells, established on collagen-coated membranes. Am J Physiol Renal Physiol. 2007;293: F476–485. doi: 10.1152/ajprenal.00363.2006 17475898 10.1152/ajprenal.00363.2006

[11] Chang SH, Chiang IN, Chen YH, Young TH. Serum-free culture of rat proximal tubule cells with enhanced function on chitosan. Acta Biomater. 2013 doi: 10.1016/j.actbio.2013.06.032 23816651 10.1016/j.actbio.2013.06.032

[12] Vatansever F, Hamblin MR. Far infrared radiation (FIR): its biological effects and medical applications. Photonics & lasers in medicine. 2012;4: 255–66. doi: 10.1515/plm-2012-0034 23833705 10.1515/plm-2012-0034 PMC3699878

[13] Inoue S, Kabaya M. Biological activities caused by far-infrared radiation. Int J Biometeorol. 1989;33: 145–150. 2689357 10.1007/BF01084598

[14] Lin YH, Li TS. The Application of Far-Infrared in the Treatment of Wound Healing: A Short Evidence-Based Analysis. J Evid Based Complementary Altern Med. 2015 doi: 10.1177/2156587215623436 26703225 10.1177/2156587215623436 PMC5871200

[15] Yu SY, Chiu JH, Yang SD, Hsu YC, Lui WY, Wu CW. Biological effect of far-infrared therapy on increasing skin microcirculation in rats. Photodermatol Photoimmunol Photomed. 2006;22: 78–86. doi: 10.1111/j.1600-0781.2006.00208.x 16606412 10.1111/j.1600-0781.2006.00208.x

[16] Herzog J, Ehrlich SM, Pfitzer L, Liebl J, Frohlich T, Arnold GJ, et al Cyclin-dependent kinase 5 stabilizes hypoxia-inducible factor-1alpha: a novel approach for inhibiting angiogenesis in hepatocellular carcinoma. Oncotarget. 2016 doi: 10.18632/oncotarget.8342 27027353 10.18632/oncotarget.8342 PMC5053636

[17] Ma SK, Joo SY, Choi HI, Bae EH, Nam KI, Lee J, et al Activation of G-protein-coupled receptor 40 attenuates the cisplatin-induced apoptosis of human renal proximal tubule epithelial cells. Int J Mol Med. 2014;34: 1117–1123. doi: 10.3892/ijmm.2014.1874 25092426 10.3892/ijmm.2014.1874

[18] Bastepe M. The GNAS Locus: Quintessential Complex Gene Encoding Gsalpha, XLalphas, and other Imprinted Transcripts. Curr Genomics. 2007;8: 398–414. doi: 10.2174/138920207783406488 19412439 10.2174/138920207783406488 PMC2671723

[19] Perez-Nanclares G, Velayos T, Vela A, Munoz-Torres M, Castano L. Pseudohypoparathyroidism type Ib associated with novel duplications in the GNAS locus. PLoS One. 2015;10: e0117691 doi: 10.1371/journal.pone.0117691 25710380 10.1371/journal.pone.0117691 PMC4339194

[20] Shoback DM, Bilezikian JP, Costa AG, Dempster D, Dralle H, Khan AA, et al Presentation of Hypoparathyroidism: Etiologies and Clinical Features. J Clin Endocrinol Metab. 2016: jc20153909 doi: 10.1210/jc.2015-3909 26943721 10.1210/jc.2015-3909

[21] Turan S, Bastepe M. GNAS Spectrum of Disorders. Curr Osteoporos Rep. 2015;13: 146–158. doi: 10.1007/s11914-015-0268-x 25851935 10.1007/s11914-015-0268-x PMC4417430

[22] Pankow K, Wang Y, Gembardt F, Krause E, Sun X, Krause G, et al Successive action of meprin A and neprilysin catabolizes B-type natriuretic peptide. Circ Res. 2007;101: 875–882. doi: 10.1161/CIRCRESAHA.107.153585 17823376 10.1161/CIRCRESAHA.107.153585

[23] Khan AA, Sandhya VK, Singh P, Parthasarathy D, Kumar A, Advani J, et al Signaling Network Map of Endothelial TEK Tyrosine Kinase. J Signal Transduct. 2014;2014: 173026 doi: 10.1155/2014/173026 25371820 10.1155/2014/173026 PMC4211299

[24] Herrera-Perez Z, Gretz N, Dweep H. A Comprehensive Review on the Genetic Regulation of Cisplatin-induced Nephrotoxicity. Curr Genomics. 2016;17: 279–293. doi: 10.2174/1389202917666160202220555 27252593 10.2174/1389202917666160202220555 PMC4869013

[25] Rathinam R, Ghosh S, Neumann WL, Jamesdaniel S. Cisplatin-induced apoptosis in auditory, renal, and neuronal cells is associated with nitration and downregulation of LMO4. Cell Death Discov. 2015;1 doi: 10.1038/cddiscovery.2015.52 26925255 10.1038/cddiscovery.2015.52 PMC4765951

[26] Terada Y, Inoue K, Matsumoto T, Ishihara M, Hamada K, Shimamura Y, et al 5-Aminolevulinic acid protects against cisplatin-induced nephrotoxicity without compromising the anticancer efficiency of cisplatin in rats in vitro and in vivo. PLoS One. 2013;8: e80850 doi: 10.1371/journal.pone.0080850 24324635 10.1371/journal.pone.0080850 PMC3855642

