# Supplementary material for: Far infrared radiation promotes rabbit renal proximal tubule cell proliferation and functional characteristics, and protects against cisplatin-induced nephrotoxicity
Source: PLoS One. 2017 Jul 17;12(7):e0180872. doi: 10.1371/journal.pone.0180872 (PMC5513434; doi:10.1371/journal.pone.0180872)
Supplement: S1 File — Exposure to FIR increases renal cell proliferation. (PDF) [file pone.0180872.s001.pdf]

Control					MEAN	SD	SE
DAY2	0.249	0.273	0.2451	0.2399	0.25175	0.014649	0.007324
DAY4	0.3954	0.4066	0.3986	0.3886	0.3973	0.007472	0.003736
DAY6	0.4109	0.308	0.5225	0.4261	0.416875	0.087809	0.043904
DAY8	0.5955	0.4451	0.4598	0.5192	0.5049	0.068369	0.034184

FIR							
DAY2	0.2542	0.2691	0.272	0.2762	0.267875	0.009571	0.004786
DAY4	0.4026	0.3898	0.418	0.4188	0.4073	0.013845	0.006923
DAY6	0.6094	0.4824	0.5334	0.6217	0.561725	0.065739	0.032869
DAY8	0.8103	0.7667	0.7258	0.8354	0.78455	0.04837	0.024185

MEAN	DAY2	DAY4	DAY6	DAY8
Control	0.25175	0.3973	0.416875	0.5049
FIR	0.267875	0.4073	0.561725	0.78455

SE	DAY2	DAY4	DAY6	DAY8
Control	0.007324	0.003736	0.043904	0.034184
FIR	0.004786	0.006923	0.032869	0.024185
