# Supplementary figures and images for: Far infrared radiation promotes rabbit renal proximal tubule cell proliferation and functional characteristics, and protects against cisplatin-induced nephrotoxicity
Source: PLoS One. 2017 Jul 17;12(7):e0180872. doi: 10.1371/journal.pone.0180872 (PMC5513434; doi:10.1371/journal.pone.0180872)

Date 20150527

Date 20150602

Date 20150815

Date 20150902

GAPDH

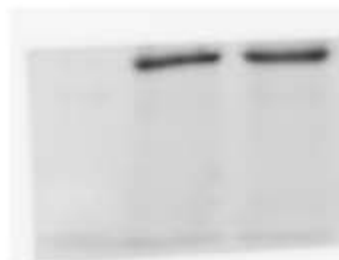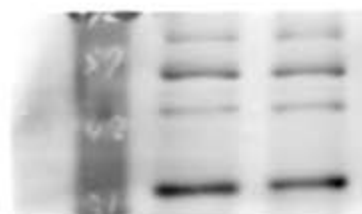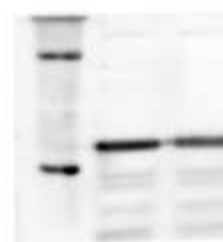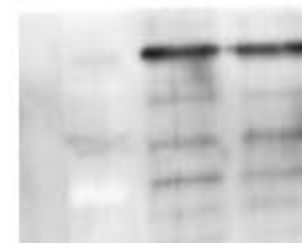

GLUT1

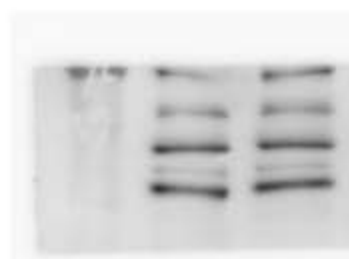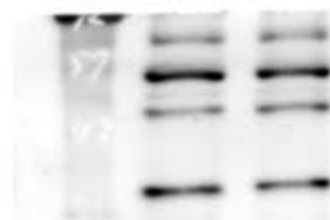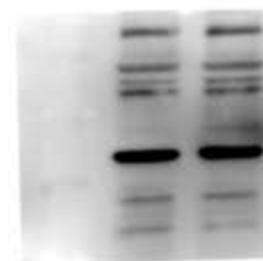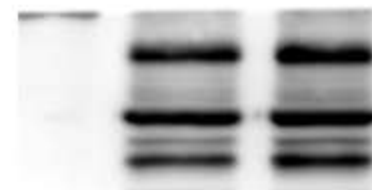

$\alpha$ -K ATPase

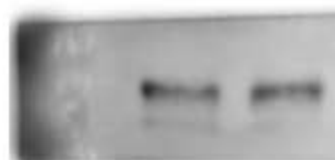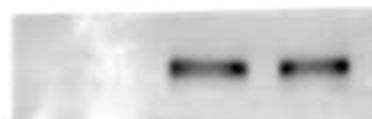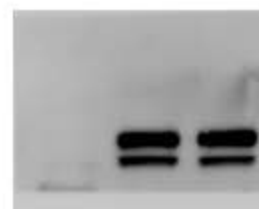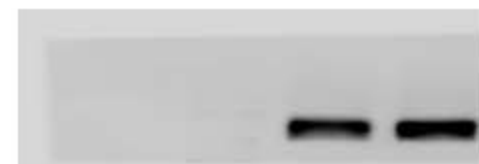

Supplement: S2 File — FIR exposure increases ATPase Na+/K+ subunit alpha 1 and GLUT1 expression. (PDF) [file pone.0180872.s002.pdf]
