# Supplementary material for: Far infrared radiation promotes rabbit renal proximal tubule cell proliferation and functional characteristics, and protects against cisplatin-induced nephrotoxicity
Source: PLoS One. 2017 Jul 17;12(7):e0180872. doi: 10.1371/journal.pone.0180872 (PMC5513434; doi:10.1371/journal.pone.0180872)
Supplement: S3 File — FIR exposure enhances the expression of CDK5R1, GNAS, NPPB, and TEK in RPTCs. (PDF) [file pone.0180872.s003.pdf]

gene primer	Position	Cq	GAPDH Cq	- ΔCT	2^-ΔCT	MEAN	SD
ATP5B	RPTC Normal	21.62	17.46	-4.16	0.055939067	0.059104	0.006896
		21.74	17.6	-4.14	0.056719947		
		21.59	17.74	-3.85	0.069348092		
		21.69	17.49	-4.2	0.05440941		
	RPTC FIR	21.31	17.22	-4.09	0.058720172	0.058117	0.009314
		21.45	17.55	-3.9	0.066985841		
		21.33	17.31	-4.02	0.061639544		
		21.42	16.95	-4.47	0.045122787		
CACNA1A	RPTC Normal						
	RPTC FIR						
CDK5R1	RPTC Normal	35.56	17.46	-18.1	3.55924E-06	3.91E-06	2.43E-07
		35.5	17.6	-17.9	4.08849E-06		
		35.65	17.74	-17.91	4.06025E-06		
		35.45	17.49	-17.96	3.92194E-06		
	RPTC FIR	32.19	17.22	-14.97	3.11588E-05	2.75E-05	3.64E-06
		32.6	17.55	-15.05	2.9478E-05		
		32.5	17.31	-15.19	2.67519E-05		
		32.37	16.95	-15.42	2.28096E-05		
GNAS	RPTC Normal	38.37	17.46	-20.91	5.07531E-07	4.95E-07	4.2E-08
		38.74	17.6	-21.14	4.32739E-07		
		38.61	17.74	-20.87	5.218E-07		
		38.37	17.49	-20.88	5.18196E-07		
	RPTC FIR	37.33	17.22	-20.11	8.83663E-07	9.23E-07	2.42E-07
		37.81	17.55	-20.26	7.96402E-07		
		36.89	17.31	-19.58	1.27595E-06		
		37.32	16.95	-20.37	7.37936E-07		
NPPB	RPTC Normal	41.53	17.46	-24.07	5.67816E-08	6.37E-08	1.02E-08
		41.2	17.6	-23.6	7.86488E-08		
		41.78	17.74	-24.04	5.79748E-08		
		41.45	17.49	-23.96	6.12804E-08		
	RPTC FIR	36.44	17.22	-19.22	1.63758E-06	1.28E-06	3.13E-07
		37.59	17.55	-20.04	9.27596E-07		
		37.07	17.31	-19.76	1.12628E-06		
		36.38	16.95	-19.43	1.41575E-06		
TEK	RPTC Normal	42.78	17.46	-25.32	2.38737E-08	2.43E-08	1.24E-08
		42.11	17.6	-24.51	4.18557E-08		
		43.63	17.74	-25.89	1.60818E-08		
		43.46	17.49	-25.97	1.52143E-08		
	RPTC FIR	39.51	17.22	-22.29	1.95003E-07	2.38E-07	7.8E-08
		39.16	17.55	-21.61	3.12422E-07		
		39.98	17.31	-22.67	1.49848E-07		
		38.65	16.95	-21.7	2.93528E-07		
GAPDH	RPTC Normal	17.46	17.46	0			
		17.6	17.6	0			
		17.74	17.74	0			
		17.49	17.49	0			
	RPTC FIR	17.22	17.22	0			
		17.55	17.55	0			
		17.31	17.31	0			
		16.95	16.95	0			

MEAN	ATP5B	CACNA1A	CDK5R1	GNAS	NPPB	TEK
Normal	0.059104129	0	3.90748E-06	4.95E-07	6.36714E-08	2.43E-08
FIR	0.058117086	0	2.75496E-05	9.23E-07	1.2768E-06	2.38E-07

SD	ATP5B	CACNA1A	CDK5R1	GNAS	NPPB	TEK
Normal	0.006896403	0	2.433E-07	4.2E-08	1.01646E-08	1.24E-08
FIR	0.00931447	0	3.6446E-06	2.42E-07	3.13088E-07	7.8E-08
