# Supplementary material for: Far infrared radiation promotes rabbit renal proximal tubule cell proliferation and functional characteristics, and protects against cisplatin-induced nephrotoxicity
Source: PLoS One. 2017 Jul 17;12(7):e0180872. doi: 10.1371/journal.pone.0180872 (PMC5513434; doi:10.1371/journal.pone.0180872)
Supplement: S4 File — Exposure to FIR protects HK-2 cells from cisplatin-induced nephrotoxicity. (PDF) [file pone.0180872.s004.pdf]

Application: Tecan i-control Tecan i-control , 1.12.4.0
Device: infinite 200 Serial number: 611000017 Serial number of connected stacker:
Firmware: V_2.02_11/06_InfiniTe (Dec 1: MAI, V_2.02_11/06_InfiniTe (Dec 13 2006/16.18.16)

Date: ########
Time: 下午 02:23:10

System RCF8-EB4F-03
User NTUH\ntuhuser
Plate Costar 96 Flat Bottom Transparent Polystyrene Cat. No.: 3361/3590/9018/3591/9017/3641/3628/3370/2507/2509/2503/9017/9018/3641/3598/3599/3585/3595/3300/3474 [COS96ft.pdfx]
Plate-ID (Stacker)

Shaking (Orbital) Duration: 5 s
Shaking (Orbital) Amplitude: 1 mm

Label: Label1
Mode Absorbance
Wavelength 570 nm
Bandwidth 10 nm
Number of Flashes 5
Settle Time 0 ms
Part of Plate C3-G10
Start Time 2016/12/2 下午 02:23:15

Temperature: 20.4 °C

<>	3	4	5	6	7	8	9	10	cisplatin (uM)
C	0.3249	0.3641	0.3815	0.368	0.3755	0.3881	0.4059	0.3816	0
D	0.2414	0.2713	0.2879	0.2821	0.2997	0.2865	0.2744	0.2504	50
E									
F									
G									

End Time: 2016/12/2 下午 02:23:30

cisplatin (uM)									mean	S.D
0	0.3249	0.3641	0.3815	0.368	0.3755	0.3881	0.4059	0.3816	0.3737	0.023534
50	0.2414	0.2713	0.2879	0.2821	0.2997	0.2865	0.2744	0.2504	0.274212	0.019648

Application: Tecan i-control	Tecan i-control , 1.12.4.0	
Device: infinite 200	Serial number: 611000017	Serial number of connected stacker:
Firmware: V_2.02_11/06_InfiniTe (Dec 1	MAI, V_2.02_11/06_InfiniTe (Dec 13 2006/16.18.16)	

Date:	########
Time:	下午 02:22:22

System	RCF8-EB4F-03
User	NTUH\ntuhuser
Plate	Costar 96 Flat Bottom Transparent Polystyrene Cat. No.: 3361/3590/9018/3591/9017/3641/3628/3370/2507/2509/2503/9017/9018/3641/3598/3599/3585/3595/3300/3474 [COS96ft.pdfx]
Plate-ID (Stacker)	

Shaking (Orbital) Duration:	5 s
Shaking (Orbital) Amplitude:	1 mm

Label: Label1

Mode	Absorbance
Wavelength	570 nm
Bandwidth	10 nm
Number of Flashes	5
Settle Time	0 ms
Part of Plate	C3-G10
Start Time	2016/12/2 下午 02:22:28

Temperature: 20.4 °C

<>	3	4	5	6	7	8	9	10	cisplatin (uM)
C	0.294	0.3495	0.4046	0.4205	0.4273	0.4162	0.4128	0.4218	0
D	0.2572	0.2499	0.2582	0.2794	0.2261	0.2656	0.281	0.2145	50
E									
F									
G									

End Time: 2016/12/2 下午 02:22:43

cisplatin (uM)									mean	S.D
0	0.294	0.3495	0.4046	0.4205	0.4273	0.4162	0.4128	0.4218	0.393338	0.047086
50	0.2572	0.2499	0.2582	0.2794	0.2261	0.2656	0.281	0.2145	0.253987	0.02358

Application: Tecan i-control   Tecan i-control , 1.12.4.0
Device: infinite 200   Serial number: 611000017   Serial number of connected stacker:
Firmware: V_2.02_11/06_InfiniTe (Dec 1:MAI, V_2.02_11/06_InfiniTe (Dec 13 2006/16.18.16)

Date: ########
Time: 下午 07:25:10

System	RCF8-EB4F-03
User	NTUH\ntuhuser
Plate	Costar 96 Flat Bottom Transparent Polystyrene Cat. No.: 3361/3590/9018/3591/9017/3641/3628/3370/2507/2509/2503/9017/9018/3641/3598/3599/3585/3595/3300/3474 [COS96ft.pdfx]
Plate-ID (Stacker)	

Shaking (Orbital) Duration:	5 s
Shaking (Orbital) Amplitude:	1 mm

Label: Label1

Mode	Absorbance
Wavelength	570 nm
Bandwidth	10 nm
Number of Flashes	5
Settle Time	0 ms
Part of Plate	A9-B12; C5-G8
Start Time	2016/12/21 下午 07:25:15

Temperature: 21.9 ˚C

<>	5	6	7	8	9	10	11	12	
A									
B									
C	0.4919	0.4765	0.4666						0
D	0.2853	0.2682	0.2352						50
E									
F									
G									

End Time: 2016/12/21 下午 07:25:27

cisplatin (uM)				mean	S.D
0	0.4919	0.4765	0.4666	0.478333	0.012749
50	0.2853	0.2682	0.2352	0.2629	0.025467

Application: Tecan i-control   Tecan i-control , 1.12.4.0
Device: infinite 200   Serial number: 611000017   Serial number of connected stacker:
Firmware: V_2.02_11/06_InfiniTe (Dec 1[illegible] MAI, V_2.02_11/06_InfiniTe (Dec 13 2006/16.18.16)

Date: ########
Time: 下午 07:24:29

System   RCF8-EB4F-03
User   NTUH\ntuhuser
Plate   Costar 96 Flat Bottom Transparent Polystyrene Cat. No.: 3361/3590/9018/3591/9017/3641/3628/3370/2507/2509/2503/9017/9018/3641/3598/3599/3585/3595/3300/3474 [COS96ft.pdfx]
Plate-ID (Stacker)

Shaking (Orbital) Duration: 5 s
Shaking (Orbital) Amplitude: 1 mm

Label: Label1
Mode   Absorbance
Wavelength   570 nm
Bandwidth   10 nm
Number of Flashes   5
Settle Time   0 ms
Part of Plate   A9-B12; C5-G8
Start Time 2016/12/21 下午 07:24:34

Temperature: 22.2 °C

<>	5	6	7	8	9	10	11	12	
A									
B									
C									
D	0.4562	0.4541	0.4774						0
E	0.1406	0.1811	0.195						50
F									
G									

End Time: 2016/12/21 下午 07:24:47

cisplatin (uM)				mean	S.D
0	0.4562	0.4541	0.4774	0.462567	0.012889
50	0.1406	0.1811	0.195	0.172233	0.028263

Application: Tecan i-control Tecan i-control , 1.12.4.0
Device: infinite 200 Serial number: 611000017 Serial number of connected stacker:
Firmware: V_2.02_11/06_InfiniTe (Dec 13 MAI, V_2.02_11/06_InfiniTe (Dec 13 2006/16.18.16)

Date: ########
Time: 下午 04:05:34

System	RCF8-EB4F-03
User	NTUH\ntuhuser
Plate	Costar 96 Flat Bottom Transparent Polystyrene Cat. No.: 3361/3590/9018/3591/9017/3641/3628/3370/2507/2509/2503/9017/9018/3641/3598/3599/3585/3595/3300/3474 [COS96ft.pdfx]
Plate-ID (Stacker)	

Shaking (Orbital) Duration:	5 s
Shaking (Orbital) Amplitude:	1 mm

Label: Label1

Mode	Absorbance
Wavelength	570 nm
Bandwidth	10 nm
Number of Flashes	5
Settle Time	0 ms
Part of Plate	C5-G9
Start Time	2017/1/26 下午 04:05:39

Temperature: 19.1 °C

<>	5	6	7	8	
C	0.2067	0.2375	0.2346	0.2318	0
D	0.1321	0.1528	0.1506	0.1449	50
E					
F					
G					

End Time: 2017/1/26 下午 04:05:51

cisplatin					mean	S.D
0	0.2067	0.2375	0.2346	0.2318	0.234633	0.014159
50	0.1321	0.1528	0.1506	0.1449	0.149433	0.009284

Application: Tecan i-control	Tecan i-control , 1.12.4.0		
Device: infinite 200	Serial number: 611000017	Serial number of connected stacker:	
Firmware: V_2.02_11/06_InfiniTe (Dec 1:	MAI, V_2.02_11/06_InfiniTe (Dec 13 2006/16.18.16)		

Date: ########
Time: 下午 04:04:08

System	RCF8-EB4F-03
User	NTUH\ntuhuser
Plate	Costar 96 Flat Bottom Transparent Polystyrene Cat. No.: 3361/3590/9018/3591/9017/3641/3628/3370/2507/2509/2503/9017/9018/3641/3598/3599/3585/3595/3300/3474 [COS96ft.pdfx]
Plate-ID (Stacker)	

Shaking (Orbital) Duration:	5 s
Shaking (Orbital) Amplitude:	1 mm

Label: Label1

Mode	Absorbance
Wavelength	570 nm
Bandwidth	10 nm
Number of Flashes	5
Settle Time	0 ms
Part of Plate	C5-G9
Start Time	2017/1/26 下午 04:04:13

Temperature: 18.9 °C

<>	5	6	7	8	
C	0.197	0.2375	0.2202	0.214	0
D	0.1434	0.1413	0.1397	0.128	50
E					
F					
G					

End Time: 2017/1/26 下午 04:04:25

cisplatin					mean	S.D
0	0.197	0.2375	0.2202	0.214	0.2239	0.016727
50	0.1434	0.1413	0.1397	0.128	0.141467	0.006902

Application: Tecan i-control	Tecan i-control , 1.12.4.0		
Device: infinite 200	Serial number: 611000017	Serial number of connected stacker:	
Firmware: V_2.02_11/06_InfiniTe (Dec 1:	MAI, V_2.02_11/06_InfiniTe (Dec 13 2006/16.18.16)		

Date: ########
Time: 下午 05:52:01

System	RCF8-EB4F-02
User	RCF8-EB4F-02\USER
Plate	Costar 96 Flat Bottom Transparent Polystyrene Cat. No.: 3361/3590/9018/3591/9017/3641/3628/3370/2507/2509/2503/9017/9018/3641/3598/3599/3585/3595/3300/3474 [COS96ft.pdfx]
Plate-ID (Stacker)	

Shaking (Orbital) Duration:	5 s
Shaking (Orbital) Amplitude:	1 mm

Label: Label1

Mode	Absorbance
Wavelength	570 nm
Bandwidth	10 nm
Number of Flashes	25
Settle Time	0 ms
Part of Plate	C3-F10
Start Time	2017/2/26 下午 05:52:06

Temperature: 18.9 ˚C

<>	3	4	5	6	7	8	9	10	
C	0.2353	0.2389	0.2383	0.2288					0
D	0.1428	0.1533	0.1514	0.1519					50
E									
F									

End Time: 2017/2/26 下午 05:52:35

cisplatin					mean	S.D
0	0.2353	0.2389	0.2383	0.2288	0.2375	0.004626
50	0.1428	0.1533	0.1514	0.1519	0.1522	0.004768

Application: Tecan i-control Tecan i-control , 1.12.4.0
Device: infinite 200 Serial number: 611000017 Serial number of connected stacker:
Firmware: V_2.02_11/06_InfiniTe (Dec 13 MAI, V_2.02_11/06_InfiniTe (Dec 13 2006/16.18.16)

Date: ########
Time: 下午 05:51:04

System	RCF8-EB4F-02
User	RCF8-EB4F-02\USER
Plate	Costar 96 Flat Bottom Transparent Polystyrene Cat. No.: 3361/3590/9018/3591/9017/3641/3628/3370/2507/2509/2503/9017/9018/3641/3598/3599/3585/3595/3300/3474 [COS96ft.pdfx]
Plate-ID (Stacker)	

Shaking (Orbital) Duration:	5 s
Shaking (Orbital) Amplitude:	1 mm

Label: Label1

Mode	Absorbance
Wavelength	570 nm
Bandwidth	10 nm
Number of Flashes	25
Settle Time	0 ms
Part of Plate	C3-F10
Start Time	2017/2/26 下午 05:51:10

Temperature: 18.8 °C

<>	3	4	5	6	7	8	9	10	
C	0.2442	0.2458	0.2413	0.2086					0
D	0.1291	0.1352	0.1341	0.1328					50
E									
F									

End Time: 2017/2/26 下午 05:51:39

cisplatin					mean	S.D
0	0.2442	0.2458	0.2413	0.2086	0.243767	0.017682
50	0.1291	0.1352	0.1341	0.1328	0.134033	0.002655
