# Supplementary material for: Far infrared radiation promotes rabbit renal proximal tubule cell proliferation and functional characteristics, and protects against cisplatin-induced nephrotoxicity
Source: PLoS One. 2017 Jul 17;12(7):e0180872. doi: 10.1371/journal.pone.0180872 (PMC5513434; doi:10.1371/journal.pone.0180872)
Supplement: S5 File — Cisplatin-induced apoptosis is blocked by FIR exposure in HK-2 cells. (PDF) [file pone.0180872.s005.pdf]

The result of flow cytometry

Control without cisplatin

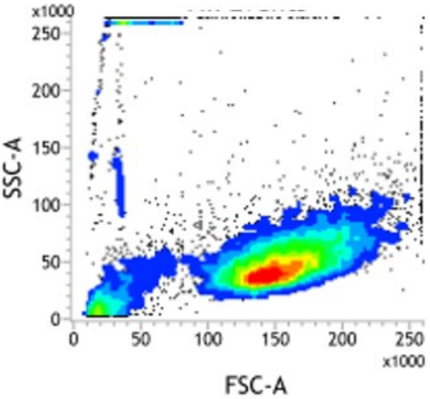

Control without cisplatin

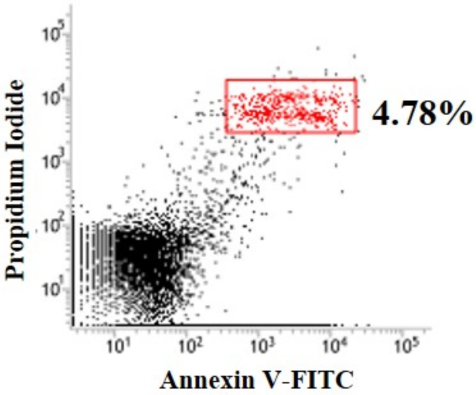

Control with cisplatin

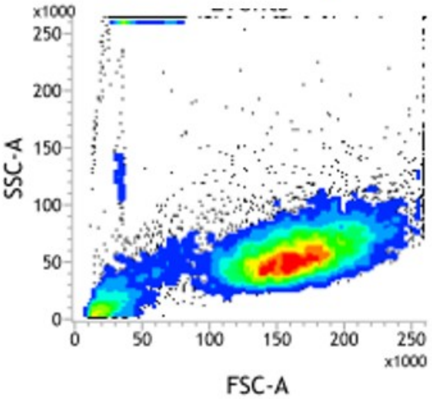

Control with cisplatin

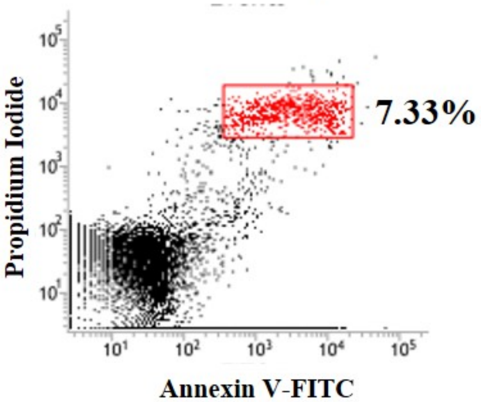

FIR without cisplatin

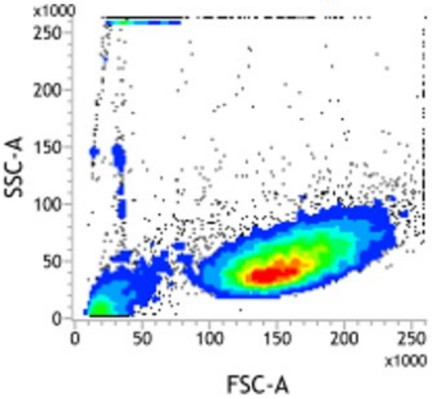

FIR without cisplatin

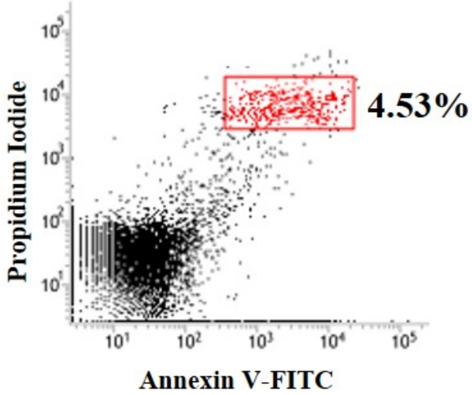

FIR with cisplatin

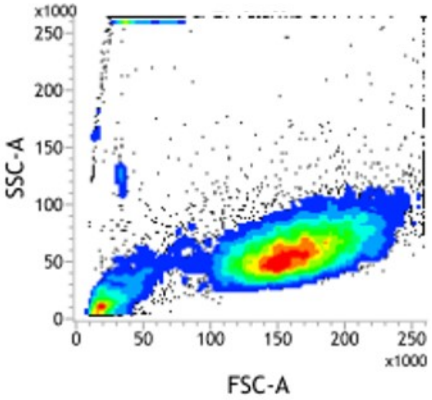

FIR with cisplatin

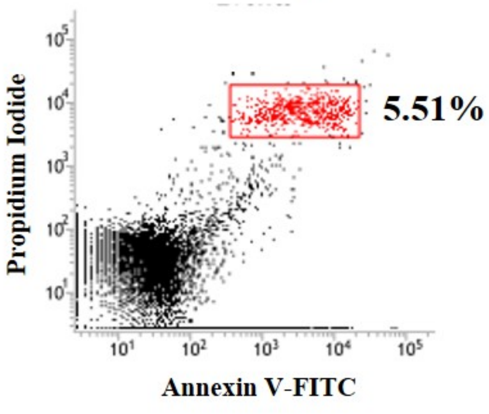

The result of flow cytometry

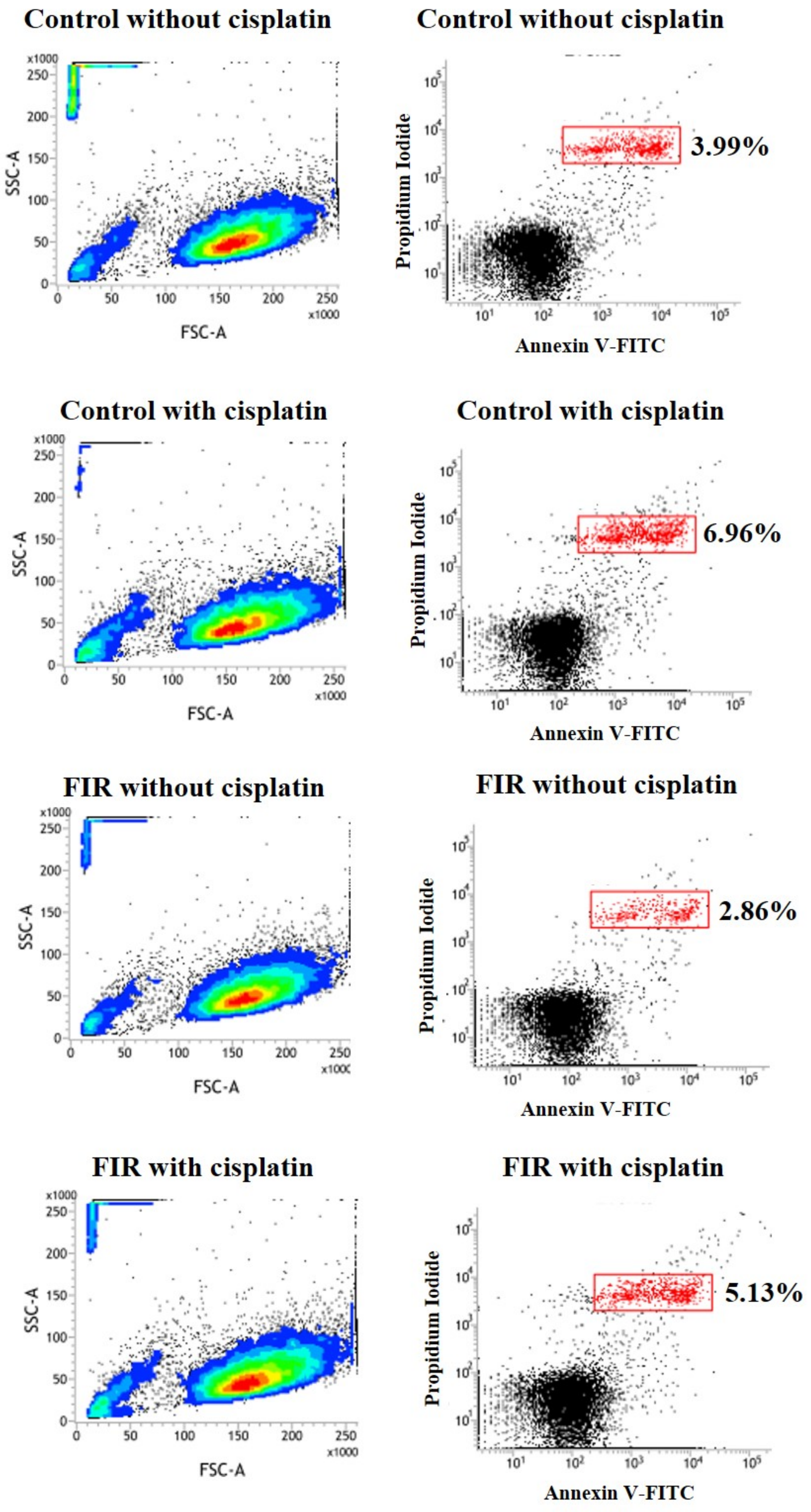

Seeding cell---->O/N---->FIR---->cisplatin 3hr---->replace medium----> O/N----> MTT

Cell number: 1x10^5/well in 6 well plate

FIR condition : High, 1hr

cisplatin concentration: 50uM

The result of flow cytometry

Control without cisplatin

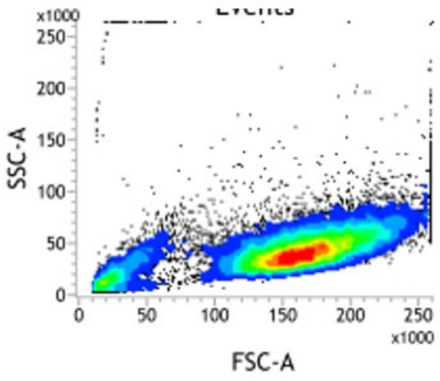

Control without cisplatin

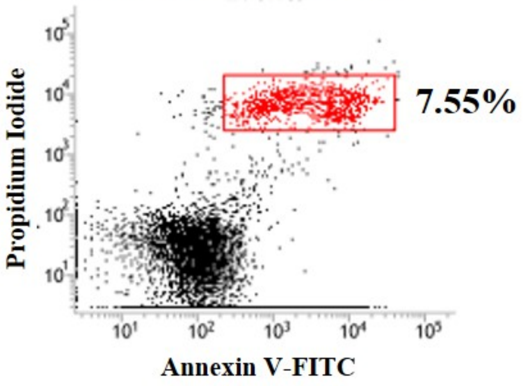

Control with cisplatin

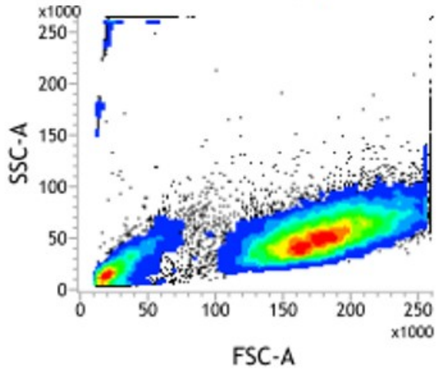

Control with cisplatin

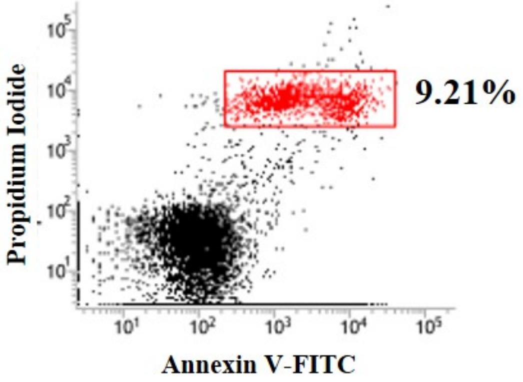

FIR without cisplatin

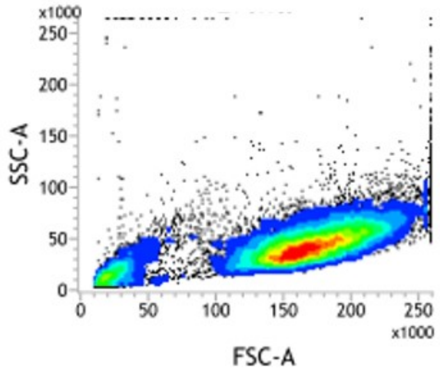

FIR without cisplatin

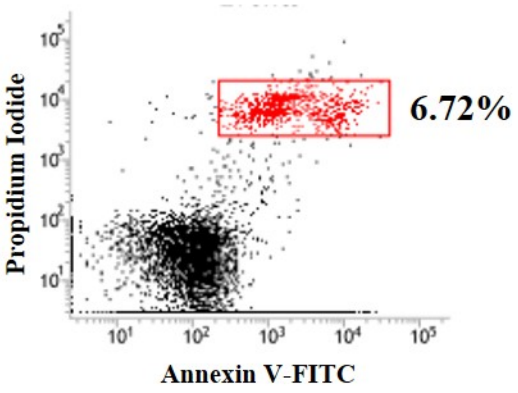

FIR with cisplatin

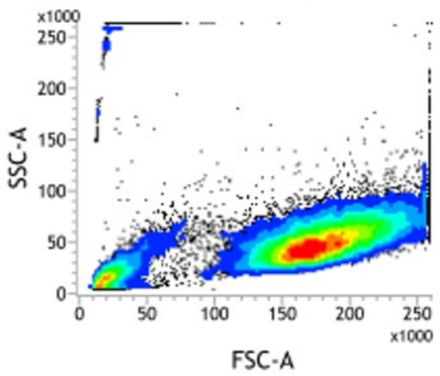

FIR with cisplatin

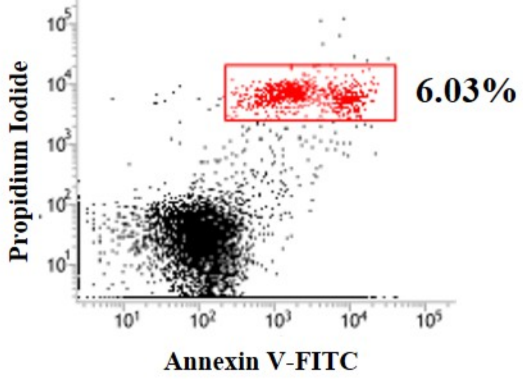

Seeding cell---->O/N---->FIR---->cisplatin 3hr---->replace medium----> O/N----> MTT
Cell number: 1x10^5/well in 6 well plate
FIR condition : High, 1hr
cisplatin concentration: 50uM
