# Supplementary material for: Far infrared radiation promotes rabbit renal proximal tubule cell proliferation and functional characteristics, and protects against cisplatin-induced nephrotoxicity
Source: PLoS One. 2017 Jul 17;12(7):e0180872. doi: 10.1371/journal.pone.0180872 (PMC5513434; doi:10.1371/journal.pone.0180872)
Supplement: S6 File — FIR exposure reduces caspase-3 activation. (PDF) [file pone.0180872.s006.pdf]

HK-2, caspase-3 assay	20170216	20170220	20170304	mean	S.D
	O.D 405 nm	O.D 405 nm	O.D 405 nm		
Control, 0uM	0.0863	0.0806	0.0821	0.083	0.002955
Control, 50uM	0.2174	0.1766	0.2135	0.2025	0.022515
FIR, 0uM	0.1221	0.164	0.1442	0.143433	0.020961
FIR, 50uM	0.157	0.1706	0.1099	0.145833	0.031853

HK-2, caspase-3 assay	20170216	20170220	20170304	mean	S.D
	normalized to 0uM	normalized to 0uM	normalized to 0uM		
Control, 0uM	1	1	1	1	0
Control, 50uM	2.519119351	2.191066998	2.600487211	2.436891	0.216743
FIR, 0uM	1	1	1	1	0
FIR, 50uM	1.285831286	1.040243902	0.762135922	1.029404	0.262016
